# Oxytocin as a physiological correlate of dyadic music therapy relationships — a randomized crossover pilot study

**DOI:** 10.3389/fnbeh.2024.1504229

**Published:** 2025-01-30

**Authors:** Paula Kristin Busse, Lutz Neugebauer, Götz Kaschubowski, Dennis Anheyer, Thomas Ostermann

**Affiliations:** ^1^Department of Psychology and Psychotherapy, Witten/ Herdecke University, Alfred-Herrhausen-Straße, Witten, Germany; ^2^Nordoff/Robbins Center for Music Therapy, Witten, Germany; ^3^Old Vicarage Special School, Kirchender Dorfweg, Herdecke, Germany; ^4^Institute for General Practice and Interprofessional Care, University Hospital Tübingen, Tübingen, Germany; ^5^Robert Bosch Centre for Integrative Medicine and Health, Auerbachstraße, Stuttgart, Germany

**Keywords:** attachment, music, music therapy, oxytocin, psychotherapy, pilot study

## Abstract

**Rationale:**

Music therapy has been in practice for years. However, the mechanism of action of music or music therapy is not well understood. It is only recently that the neuroendocrinological basis of therapeutic relationships has become the subject of growing research interest. The aim of this pilot study (Clinical Trial No: DRKS00035174) is to investigate whether oxytocin is usable and feasible as a biomarker of attachment to demonstrate the development of therapeutic alliance between therapist and patient in a dyadic music therapy setting.

**Methods:**

In a single-measure crossover design, children aged 6–12 years from a special school for social and emotional disorders, were randomly with either music therapy followed by a waiting list control group that performed silent work, or vice versa. The respective interventions were conducted on the school premises on different days over a period of 1 month. The primary outcome was salivary oxytocin, with tests performed immediately before and after each 30-min intervention.

**Results:**

Thirty-two children were included in the study, resulting in *n* = 16 children per allocation sequence. During the implementation of the study, difficulties were encountered with protocol adherence both in terms of the duration of the music therapy and the implementation of the silent work in the control group. There were no dropouts, however, only 28 children were included in the final data analysis as two participants in each group were excluded due to large fluctuations in oxytocin levels. Between-group comparison and within-group comparisons showed no significant changes in oxytocin levels. However, the music therapist showed a significant increase in oxytocin levels in the before after measurement. No side effects or adverse events were reported during the trial.

**Conclusion:**

The findings indicated a responsiveness of oxytocin to musical stimulation. Although feasibility of oxytocin measurement was clearly demonstrated, evaluation of the results is difficult against the background of many remaining questions regarding individual and contextual factors influencing the oxytocinergic system. Moreover, the clinical significance of changes in oxytocin levels remains a topic for further research to better understand the role of oxytocin in the attachment formation between therapist and patient in music therapy.

## Introduction

### Attachment theory

The foundations of attachment theory were established in the 20th century by the pediatrician John Bowlby and the developmental psychologist Mary Ainsworth (Ainsworth and Bowlby, 1991; Bretherton, 1992). In their work, attachment is defined as an independent neurobiological system that develops through interaction between the child and the caregiver, providing the child with a sense of protection and a secure base from which to explore and return to (Benoit, 2004; Chambers, 2017). According to attachment theory, this creates an inner working model of relationships, which can result in four different attachment styles: secure, insecure-avoidant, insecure-resistant and insecure-disorganized (Archer et al., 2015; Shemmings, 2006).

Over the years, a large body of literature has accumulated investigating the link between attachment style and mental or physical health outcomes as well as social functioning and affect regulation (Groh et al., 2017; Pietromonaco and Beck, 2019; Widom et al., 2018). Compared to secure attachment styles, insecure attachment styles are associated with a range of emotional and behavioral problems, including depression (Simon et al., 2019; Warfa et al., 2014), anxiety (Manning et al., 2017; Nielsen et al., 2017), suicidal behavior (Falgares et al., 2017; Naifeh et al., 2024) as well as the risk and severity of drug addiction (Strathearn et al., 2019; Valizadeh et al., 2017). It is further a greater risk for the development of chronic pain (Romeo et al., 2017), increased pain perception (Peñacoba et al., 2018), and an overall reduced quality of life (Bodner and Cohen-Fridel, 2010; Karveli et al., 2023).

Attachment styles primarily develop in early childhood in relation to primary caregivers and often show a certain stability over time. However, they can also change during life in relation to subsequent attachment figures, thereby alter a persons’ sense of attachment security (Mikulincer et al., 2013; Pinquart et al., 2013). According to Bowlby, a variety of people can serve as attachment figures if they meet a number of conditions: 1. maintain proximity, 2. represent a safe haven physically and emotionally, and 3. provide a secure base from which to explore (Bowlby, 1982). In this way, a therapist can also provide a patient with a secure base and strengthen the feeling of attachment security (Mikulincer et al., 2013).

Although the concepts of ‘attachment’ and ‘bonding’ are often used interchangeably in the literature, the difference should be articulated at this juncture. While both theories deal with the formation of relationships, bonding primarily refers to physical closeness, especially skin-to-skin contact, during a certain period after birth, which is primarily intended to serve the acceptance of the child by the parents (Ettenberger et al., 2021).

### Methods for measuring attachment

Various methods have been used to measure attachment, including interviews like the Adult Attachment Interview (AAI) (George et al., 1985), self-reports (Hazan and Shaver, 1987), scales and questionnaires (Ravitz et al., 2010), behavioral observation as in the Strange Situation Test developed by Mary Ainsworth (Van Rosmalen et al., 2015) and, more recently, the collection of biomarkers such as oxytocin (Feldman, 2012; Tops et al., 2007). To date, self-reports have been used predominantly to determine therapy outcomes, but with large numbers of patients seeking psychotherapy, there is a growing demand to complement subjective reports with objective measures such as biomarkers (Atzil-Slonim et al., 2022). However, particularly the investigation of underlying mechanisms of psychotherapy and the neurobiological basis of human attachment, is still in its early stages (Zilcha-Mano et al., 2020).

Evidence of endocrine synchrony in periods of bond formation has previously been found between parents and their children as well romantic partners (Djalovski et al., 2021; Feldman et al., 2011; Schneiderman et al., 2014; Ulmer-Yaniv et al., 2016). This raises the question of whether such a synchronization process might also occur between a therapist and his patient and, moreover, whether this hormonal attunement might correlate with the success of a therapy (Zilcha-Mano et al., 2020).

In a meta-analysis, (Flückiger et al., 2018) showed that therapeutic alliance and thus psychotherapy as an attachment-based intervention is one of the most reliable predictors of therapeutic change. Therefore, linking attachment theory with oxytocin as a potential biomarker for attachment formation, i.e., the therapeutic alliance is ostensibly promising and of growing interest (Palmieri et al., 2021; Striepens et al., 2011).

### Oxytocin as a potential biomarker for attachment

Oxytocin is a peptide hormone synthesized in the supraoptic and paraventricular nuclei of the hypothalamus. It is released both centrally and peripherally (Harvey, 2020) in situations of sensory stimulation such as skin-to-skin contact, food intake, labor and breastfeeding (Uvnäs-Moberg et al., 2015). Increasing research in oxytocin is currently been driven by its effects on social interaction in humans (Bartz et al., 2011; Erdozain and Peñagarikano, 2020) in particular in psychotherapy research. According to Baskaran et al. (2017) oxytocin has an important mediating role in human social–emotional functioning.

To date, there are only a few studies in which oxytocin levels have been measured before and after a psychotherapy session, so that overall little evidence is available in this area (Zilcha-Mano et al., 2020). Previous studies have primarily investigated the value of oxytocin as a biomarker for treatment outcomes in patients with depression. However, these studies support the hypothesis that oxytocin reactivity es treatment response. In several studies, oxytocin levels were measured in patients only, therapists only, or patients and therapists before and after treatment. Results showed that oxytocin synchrony between therapist and patient was associated with effective treatment (Zilcha-Mano et al., 2021), oxytocin plasma levels at baseline predicted improvement in self-rated depression (BDI-II-scores) (Jobst et al., 2018), therapists’ oxytocin response predicted reduction in patients’ depressive symptoms in the next session (Fisher et al., 2023), and oxytocin reactivity was associated with improvement in depressive symptoms (Atzil-Slonim et al., 2022).

Thus, the question remains to what extent hormonal changes are predictive and indicative of psychotherapeutic changes (Fischer and Zilcha-Mano, 2022). It is hypothesized that baseline levels of oxytocin represent the trait-like effects and could therefore act as a biomarker for the extent to which people are receptive to a particular treatment, i.e., baseline levels may be predictive. On the other hand, changes in oxytocin levels, the state-like-effects, over the course of a treatment may function as a biomarker for the degree of synchronization between therapist and patient, i.e., changes in oxytocin levels may be indicative (Zilcha-Mano et al., 2020).

Beyond this, there is a growing field of research dedicated to oxytocin administration and psychotherapy. The rationale here is that exogenously supplied oxytocin may act as a therapeutic agent itself or as a catalyst improving therapeutic alliance and may thus be used as an augmenting agent in psychotherapy (Koch et al., 2014; Olff et al., 2010; Zilcha-Mano et al., 2020). Although a number of studies show promising preliminary results regarding reported improvements in therapeutic outcomes, the evidence is not yet conclusive and there is still a lack of large-scale studies to substantiate the results obtained so far (Ellenbogen et al., 2024; Grossman-Giron et al., 2023; Hertenstein et al., 2021; MacDonald et al., 2013).

Altered oxytocin levels have been previously reported in a number of psychiatric disorders, including autism spectrum disorder (Huang et al., 2021; John and Jaeggi, 2021), borderline personality disorder (Bertsch et al., 2013; Carrasco et al., 2020), post-traumatic stress disorder (Carmassi et al., 2021; Donadon et al., 2018), schizophrenia (Hernández-Díaz et al., 2021; Strauss et al., 2019), social anxiety (Schneider et al., 2021) as well as in children with adverse childhood experiences (Ellis et al., 2021). Nevertheless, a meta-analysis by (Ferreira and Osório, 2022) showed equivocal results regarding altered oxytocin levels in psychiatric patients compared to healthy controls. Yet difficulties in forming relationships are characteristic of these disorders, which may also result in a lower-quality therapeutic alliance (Fischer and Zilcha-Mano, 2022). In this regard, the stimulation of endogenous oxytocin release, as possibly in music therapy, or the exogenous supply of oxytocin, as in previous studies with nasally applied oxytocin, is a promising therapeutic approach in this area of medicine (Striepens et al., 2011).

### Music therapy

Music therapy as defined by the American Music Therapy Association is “the clinical and evidence-based use of music interventions to achieve individual goals within a therapeutic relationship by a credentialed professional who has completed an approved music therapy program” (American Music Therapy Association, 2024). Therefore, music therapy uses various components of music such as melody, harmony, rhythm, timbre and pitch to support the development of a therapeutic relationship between therapist and patient (Hanson-Abromeit, 2015; Mössler et al., 2019). In this process, music therapy can consist of active or passive music interventions as well as a mixture of these qualities and may be provided both individually and in groups (Lynch et al., 2021; Wheeler et al., 2003). While music therapy requires extensive training and knowledge of musical skills on the part of the therapist, no prior musical training or musical skills are needed at the client’s site. Thus, music therapy can be used in a variety of clinical contexts for patients with physiological, psychological, spiritual, cognitive, social and behavioral challenges (Dileo and Bradt, 2009).

Music therapists use a broad repertoire of musical interventions and cover a wide range of indications across the entire lifespan of people in numerous outpatient and inpatient facilities (Kern and Tague, 2017). Target groups for music therapy include among others premature infants (Loewy et al., 2013; Standley, 2002), patients with depression and/or anxiety (Belski et al., 2022; Jasemi et al., 2016; Zhao et al., 2016), sleep disorders (Tang et al., 2022; Wang et al., 2014), dementia (Lam et al., 2020; Ueda et al., 2013), stroke (Liu et al., 2022; Wang et al., 2021), mental disorders (Gold et al., 2009), post-traumatic stress disorder (Baker et al., 2018), chronic pain (Hsu et al., 2022; Korhan et al., 2014) as well patients with cancer or in palliative care (Köhler et al., 2020; Pérez-Eizaguirre and Vergara-Moragues, 2021).

The benefits of music therapy and music’s effect on mood (Dóro et al., 2017; Raglio et al., 2015), language (Liu et al., 2022), communication and social skills (Shi et al., 2024) as well as motor, cognitive, psychological and emotional functioning (Fujioka et al., 2018; Weller and Baker, 2011) have been the subject of considerable research. And although the psychophysiology of music therapy has been examined, e.g., on its impact on the experience of pain (Arnold et al., 2024), the full landscape of mechanism of action of music therapy responsible for these outcomes is not well understood (Brancatisano et al., 2020). Indeed, music therapy has long lacked standard research tools and a common methodology, and in the past only gradually the way has been paved for evidence-based music therapy (Aldridge, 1994; Hillecke et al., 2005). Today, evidence based music therapy research includes translational research approaches such as investigations of neural mechanism and activities involved in musical perception (Thaut et al., 2021; Chen et al., 2022).

In this respect, neuroendocrinology holds great potential for enabling scientific progress in the research field of music therapy as it provides objective and reproducible results.

### Music, music therapy and oxytocin

To a considerable extent, the brain regions responding to interactive prosocial tasks overlap with those activated when making or listening to music (Harvey, 2020). It is therefore possible that the biology of oxytocin mirrors at least parts of the positive and diverse effects of music (Keeler et al., 2015).

Overall, only a few studies have investigated the relationship between oxytocin and music. In a systematic review, (Busse et al., 2024) examined studies that measured oxytocin levels before and after a music intervention. Due to highly differing study designs, study quality, sample sizes and music interventions, comparability between the studies was limited, making further studies with comparable music interventions necessary to obtain clear results. Inconclusive findings suggest that the change in oxytocin levels depends on various individual and contextual factors such as music preferences and social environment, which need to be controlled for in further studies. There was only one study included in the review involving music therapy as a music intervention. Neither the within-subject comparisons nor the comparisons between groups, with the control group undergoing a home exercise program, showed significant changes in oxytocin levels (Palumbo et al., 2022).

Combining the previously described three elements of therapy, oxytocin and music, this pilot study investigates the mediating role of oxytocin in the therapeutic synchronization process in a music therapy setting. The aim of this study is therefore to examine whether the responsiveness of biomarkers to musical stimulation can be shown in music therapy, i.e., an increase in oxytocin levels in both therapist and patient. In this way, the development of a therapeutic alliance via the biomarker oxytocin is to be demonstrated.

## Materials and methods

The study was approved by the ethics committee of the Witten/Herdecke University (No. 122/2023) and is reported based on the CONSORT 2010 guidelines and the extension for randomized crossover trials. The music therapy intervention was described according to the reporting guidelines for music interventions (Robb et al. 2011).

### Participants

Twenty-nine boys and three girls were recruited from the Old Vicarage school (“Altes Pfarrhaus”), Herdecke, Germany, a special school for children with social and emotional disorders.

The inclusion criteria for the study consisted of.

1. Children aged 6 to 12 years with social–emotional disorders and
2. The presence of a confirmed special educational need in social–emotional development based on the school assessment procedure.

The exclusion criteria were as follows.

1. The presence of cognitive, intellectual, or physical impairments that could be the cause of the social disorders,
2. A causality of the social problems in a circumscribed partial performance disorder and.
3. A concurrent participation in another clinical study or completion of participation in such a study less than 6 months ago.

The parents or legal representatives gave informed consent for their children to be involved in the study, so that ultimately all students (*n* = 32) from first to fourth grade who were attending the special school at the given time were recruited for participation.

### Procedure

A single-measure cross-over design was used for both within-subject and between-condition comparisons.

We opted for a crossover design as it allowed a small sample size to demonstrate an effect and, due to its very short half-life of <10 min in the blood (Chard et al., 1970; Leng and Sabatier, 2016), oxytocin was very well suited to carry out the two interventions in the same collective. Another consideration in favor of a crossover design was the fact that the primary therapeutic goal of music therapy for social and emotional disorders was to improve symptomatology, i.e., social functioning, so no causal change was anticipated between interventions. Further, no short-term change in the characteristic of the known social and emotional disorders was expected after a single exposure to either of the interventions, which potentially could have led to side effects, withdrawal from the study or confounding of the results. A disadvantage inherent in the design of the intervention groups was the impossibility of blinding, which had to be made an allowance for, assuming it would not have a decisive effect on the biomarker collected.

As we had no data on the expected difference between the groups, and, in addition, no estimates of within-participant variability were available in the literature for this particular study population, we used a moderate effect size of d = 0.55 to calculate the sample size. With an alpha error of 5% and a power of 80%, this resulted in a sample size of *n* = 28.

Before school started, participants of each class were randomly assigned to an allocation sequence by drawing lots: sequence one had music therapy sessions first and silent work sessions after, while sequence two had silent work sessions first followed by the music therapy sessions. The sequence was generated upon commencement of the study by the music therapist. Thus, each participant received a 30-min music therapy session as well as a 30-min silence work session, resulting in an allocation ratio of 1:1. There was at least 1 day, i.e., 24 h, between the two interventions, so that the washout phase can be considered long enough, and carryover effects can be safely excluded due to the short half-life of oxytocin.

### Music therapy

All children were already familiar with music therapy from group and individual settings in their everyday school life as music therapy is an integral part of the school program.

Music therapy was provided in the school hall which is a rectangular room with an area of 50 m^2^. As it is architecturally located in a corner of the school, it has five large window panels on two walls, flooding the room with natural daylight. The instruments are positioned so that the children face diagonally into the room while playing, with their backs to the windows. This arrangement avoids visual distractions. To match the historical building ensemble (constructed in 1820), the walls are finished with clay plaster, which creates a pleasant indoor climate and prevents temperatures from exceeding 21°C, even during warm outdoor conditions. The room is fitted with a parquet floor and the walls are painted in a pastel terracotta tone. An acoustic ceiling and curtains on the windows help prevent excessive reverberation.

The music therapy sessions are based on the Nordoff-Robbins approach (Aigen, 2014) and were always provided by the same music therapist, who was already known to the children in advance. The room is equipped with a grand piano and instruments from the orchestra percussion section such as drums. All instruments can be played by children without prior knowledge or instrumental training. They can improvise freely, setting essential musical parameters such as tempo, rhythm, volume, intensity and timbre (Neugebauer and Ostermann, 2024). Each improvisation included a four-hand piano improvisation (sometimes with spontaneous singing) and a joint improvisation by the piano (therapist) and drum cymbal (played by the child with sticks or mallets). During the piano improvisation, the child sits in the treble, while the drum and cymbal are positioned to the right in extension of the keyboard at a distance of about 1 meter from the piano. This allows eye contact during the entire improvisation.

In the control condition, the children were located in a room on their own and could choose to either paint, read, write or play in silence.

### Collection of saliva samples

Saliva samples were taken by a medically trained person before and after each session on the premises of the special school. In addition to the students, saliva samples were also taken from the music therapist before and after each music therapy session (*n* = 32). The interventions and sampling were conducted during regular school hours at identical times between 8 and 10 a.m. in order to avoid a potential distortion due to diurnal cycling of oxytocin (Forsling, 2000; Kerem and Lawson, 2021) and thus allowing the total data collection to be completed within a month.

### Measures

#### Oxytocin

As primary and exclusive outcome measure in this trial, salivary oxytocin levels were measured before and after music therapy or the control group. After collection with the Salivette® Cortisol from Sarstedt (item no. 51.1534.500), the saliva samples were frozen and stored at −20°C in a designated cooling device in the special school until analysis. Once all samples had been taken, they were transported on dry ice to the biopsychological laboratory in Dresden to ensure maintenance of the cold chain. Salivary oxytocin levels were measured using a commercially available ELISA with high sensitivity (Tecan, Hamburg, Germany; catalog number RE52331) without prior extraction. Following the laboratory’s recommendations, a total of 20% of the measurements were performed in duplicate to verify analytical quality. As reported by the laboratory, the intra- and interassay coefficients of variance were below 9%. Salivary oxytocin levels were excluded from data analysis, if differences of pre and post measures exceeded 30 pg./mL.

#### Data analysis

In this study, demographic data were routinely analyzed using descriptive statistics to summarize the key characteristics of the two groups. Comparisons between groups were performed using chi-square tests for categorical variables and t-tests or Mann–Whitney U tests for continuous variables, depending on the data distribution. These analyses ensured that any baseline differences between the groups were accounted for, minimizing potential confounding factors.

Between-group treatment outcomes were evaluated using a two-factor analysis of variance (ANOVA) to assess differences between the groups. An effect size estimate (Hedge’s g) was calculated to determine the clinical relevance of changes in oxytocin levels.

Within-group comparisons, subgroup analyses, and the evaluation of the music therapist’s oxytocin levels were performed using paired t-tests. Means, standard-deviations and corresponding 95%-confidence intervals were calculated. Additionally, assumptions for normality and homogeneity of variances were rigorously tested using Shapiro–Wilk and Levene’s tests, respectively, to ensure the validity of the statistical tests applied. In cases where these assumptions were violated, non-parametric alternatives were considered to maintain the robustness of the findings. All statistical analyses were conducted with a significance level set at *p* < 0.05, with adjustments made for multiple comparisons using the Bonferroni correction to control for Type I error. Data were analyzed with SPSS software version 28.

## Results

### Baseline characteristics

**Figure 1:**
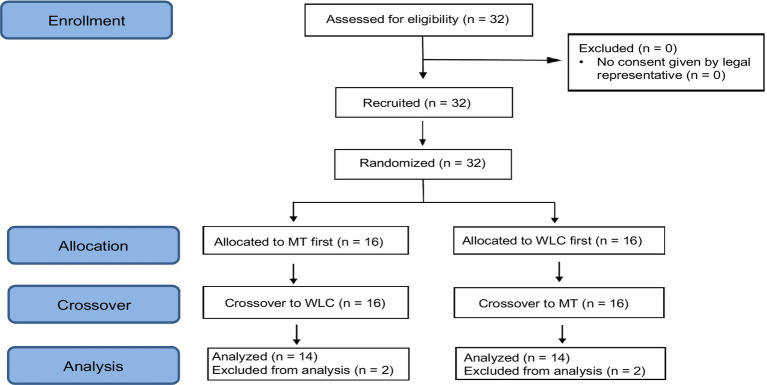
Flow of study participants. [MT = music therapy; WLC = waitlist control].

Of the 32 children who participated in the study, the results of 28 children were included in the final data analysis, as two participants in each group were excluded due to large fluctuations in oxytocin levels (Delta Ox >30 pg./mL) (Figure 1).

The remaining 28 participants had a mean chronological age of 8.7 years (SD = 1.3, range 6–11 years), including 25 boys and 3 girls. Most of the children included in the study were pre-diagnosed with multiple developmental and behavioral disorders, the most common being pervasive developmental disorders (F84) (*n* = 14), hyperkinetic disorders (F90) (*n* = 12), specific developmental disorders of speech and language (F80) (*n* = 8,), specific developmental disorder of motor function (F82) (*n* = 7), emotional disorders with onset specific to childhood (F93) (*n* = 5), disorders of social functioning with onset specific to childhood and adolescence (F94) (*n* = 5) and conduct disorders (F91) (*n* = 3). In the number of diagnoses given here, a child may have several diagnoses within one category, e.g., both an expressive and a receptive language disorder (F80.1 and F80.2). To ensure clarity, these diagnoses have been combined into superordinate groups. Concomitant pharmacological treatment with one or more medications was present in in overall nine children, including methylphenidate (*n* = 6, 18.8%), lisdexamphetamine (*n* = 3, 9.4%), risperidone (*n* = 3, 9.4%) and aripiprazole (*n* = 1, 3.1%). The respective diagnoses and medications for each child were made available from the school files. Sociodemographic and clinical characteristics are shown in Table 1.

**Table 1:** Sociodemographic and clinical data of participants (Group 1: MT first, Group 2: WLC first).

	Group 1 (*n* = 14)	Group 2 (*n* = 14)	Total (*n* = 28)	*p*-value
Age				
Mean ± SD	8.6 ± 1.3	8.8 ± 1.4	8.7 ± 1.3	0.679
Median	8	9	8	
Min	7	6	6	
Max	11	11	11	
Gender				
Male	14 (100.0%)	11 (78.6%)	25 (89.3%)	0.222
Female	0 (0.0%)	3 (21.4%)	3 (10.7%)	
Medication				
Yes	5 (35.7%)	4 (28.6%)	9 (32.1%)	1.000
No	9 (64.3%)	10 (71.4%)	19 (67.9%)	
Autism				
Yes	7 (50.0%)	7 (50.0%)	14 (50.0%)	1.000
No	7 (50.0%)	7 (50.0%)	14 (50.0%)	
Type of music therapy				
STT1	7 (50.0%)	2 (14.3%)	9 (32.1%)	0.133
STT2	0 (0.0%)	2 (14.3%)	2 (7.1%)	
P	4 (28.6%)	7 (50.0%)	11 (39.3%)	
LTT	3 (21.4%)	3 (21.4%)	6 (21.4%)	
Duration of music therapy				
Mean ± SD	22.07 ± 4.32	24.00 ± 6.81	23.04 ± 5.68	0.379
Median	22.5	23	23	
Min	12	12	12	
Max	30	35	35	

P: probationary sessions (1st-5th session). STT1: short-term therapy (1st–12th session). STT2: short-term therapy (13th-24th session). LTT: long-term therapy (>25 sessions).

At the beginning of the study, the children had already participated in music therapy to varying degrees as part of the school program. Accordingly, 11 children had already had probationary sessions, a further 11 children were in short-term therapy (STT1/STT2) and the remaining 6 children were in long-term therapy (> 25 sessions).

The duration of the music therapy varied between 12 and 35 min, as the children could not always be encouraged to participate for the entire duration of the therapy.

### Outcome measures

**Figure 2:**
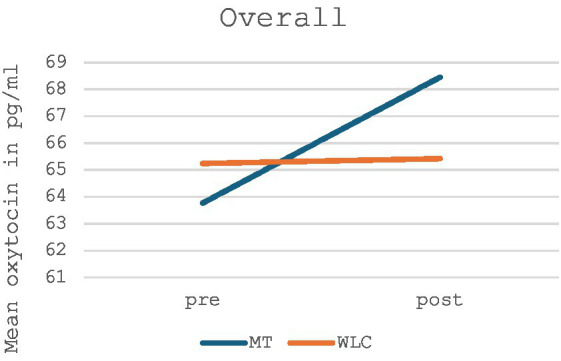
Mean oxytocin levels in music therapy [MT] compared to the waiting list control group [WLC] in the pre-post measurement for the total sample (*n* = 28).

The treatment outcome was calculated using a two-factor analysis of variance (ANOVA), with the between-group comparison, namely music therapy and control group, showing no significant changes in oxytocin levels (*p* = 0.306, *F* = 1.089). However, the changes in oxytocin levels in the music therapy group showed a non-significant increase in oxytocin levels, while the control group showed no change in oxytocin levels in the before-after measurement. Figure 2 shows the oxytocin levels for the music therapy group compared to the waiting list control group for the total sample; Table 2 provides a list of the exact values.

**Table 2:** Shown are the measured mean oxytocin levels with standard deviation, 95% confidence intervals, *p*-values for comparisons within and between the groups, *F*-values and the effect size (Hedges’ g) before and after music therapy [MT] or the waiting list control group [WLC] for the total sample, group 1, group 2, the music therapist and the subgroups of children with autism spectrum disorder [ASD] and those with medication.

		*N*	Mean (Oxytocin pg./ml)	*p* value (within groups)	SD	95% CI		Between group *p* value F Hedges’ g [95% CI]
						Lower limit	Upper limit	
Total sample	Pre MT	28	63.77	0.075	21.01	55.62	71.92	*0.306* *1.089* *0.192* *[−0.174; 0.554]*
	Post MT	28	68.45		23.34	59.4	77.5	
	Pre WLC	28	65.24	0.943	22.69	56.44	74.03	
	Post WLC	28	65.42		19.27	57.95	72.89	
Group 1	Pre MT	14	70.94	0.154	24.76	57.37	84.51	0.356
	Post MT	14	76.91		27.63	61.78	92.04	0.914
	Pre WLC	14	72.97	0.796	27.56	66.06	79.88	0.241
	Post WLC	14	71.78		19.21	61.26	82.3	[−0.265; 0.737]
Group 2	Pre MT	14	56.6	0.320	13.9	48.99	64.21	0.689
	Post MT	14	59.99		14.62	52.0	67.99	0.168
	Pre WLC	14	57.5	0.559	13.43	50.14	64.86	0.103
	Post WLC	14	59.06		17.74	49.34	68.78	[−0.292; 0.596]
Music therapist	Pre MT	28	91.2	0.006	29.11	79.93	102.48	-----
	Post MT		107.18		26.96	96.73	117.64	
ASD	Pre MT	14	63.69	0.360	24.05	49.8	77.58	-----
	Post MT		67.3		26.01	52.29	82.31	
Medication	Pre MT	9	70,85	0.192	23,75	53.64	89.06	-----
	Post MT		76,99		28,20	55.37	98.61	

Comparisons between the groups were performed using two-factorial analysis of variance (ANOVA), comparisons within the groups using the T-test for paired values.

**Figure 3:**
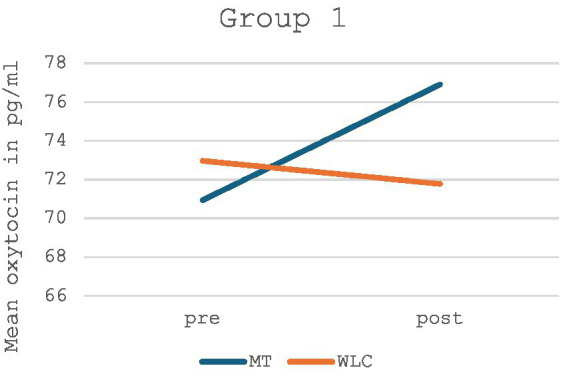
Mean oxytocin levels in music therapy [MT] compared to the waiting list control group [WLC] in the pre-post measurement for Group 1 (*n* = 14).

When looking at Group 1, there was a non-significant increase in oxytocin levels in the music therapy group and an equally non-significant decrease in oxytocin levels in the control group (*p* = 0.356, *F* = 0.914). A graphical depiction of Group 1 can be found in Figure 3, the detailed values in Table 2.

**Figure 4:**
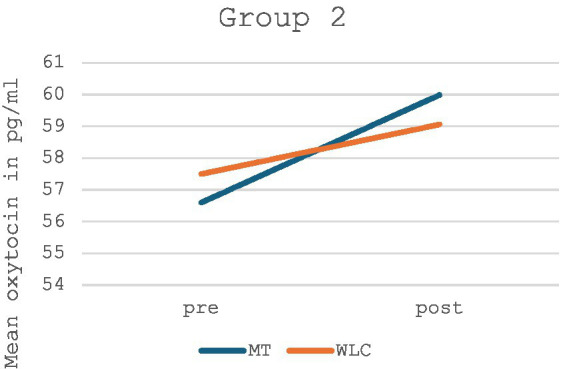
Mean oxytocin levels in music therapy [MT] compared to the waiting list control group [WLC] in the pre-post measurement for Group 2 (*n* = 14).

In contrast, Group 2 likewise showed a non-significant increase in oxytocin levels in the music therapy group, however, there was also an increase in oxytocin levels in the control group (*p* = 0.689, *F* = 0.168). An illustration and values for Group 2 can be found in Figure 4 and Table 2.

When comparing within groups using a paired-samples *t*-test, also no significant changes in oxytocin levels were found in either music therapy [*p* = 0.075, Hedges’ g = −0.340 CI95% (−0.708; 0.034)] nor the control group [*p* = 0.943, Hedges’ g = −0.013 CI95% (−0.373; 0.347)].

Nonetheless, the assessment of the within-group comparisons shows that the one-tailed *p*-value (one-tailed *p* = 0.038), in contrast to the two-tailed p-value (*p* = 0.075) for the music therapy group, is indeed significant.

In addition the effect of potentially oxytocin-level altering medication such as methylphenidate (*n* = 6, 18.8%) and lisdexamphetamine (*n* = 3, 9.4%) as described by Levi-Shachar et al. (2020) was analyzed. And although a moderately higher effect within this group was found [Hedges’ g = −0.429; CI95% (−1.042; 0.208); *p* = 0.192], it failed being significant between the groups when controlling for it (*F* = 0.635; *p* = 0.433). Moreover, there is evidence from a recent meta analysis, that children with autism have altered oxytocin levels (John and Jaeggi, 2021). As half of the children were diagnoses with Autism Spectrum Disorders (ASD), this could also have affected the results. Again, however, *post hoc* analysis of the results found an effect within the group [Hedges’ g = −0.239; CI95% (−0.735; 0.267); *p* = 0.360] but no significant difference between the groups when controlled for autism (*F* = 0.42; *p* = 0.840). All results of within-group comparisons are listed in Table 2.

**Figure 5:**
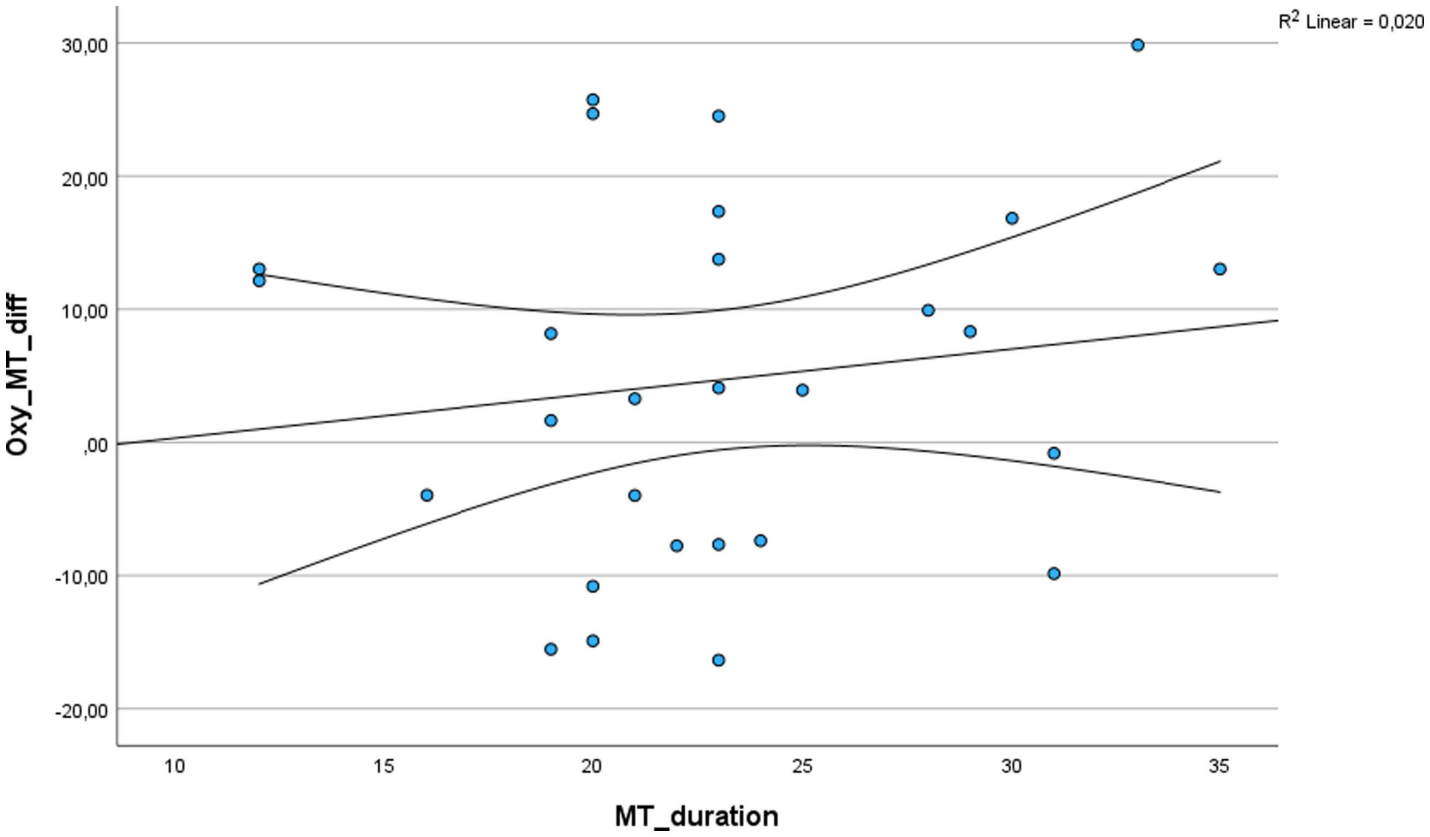
Effect of MT duration on measured oxytocin difference; MT: Music therapy.

As shown in Figure 5, the duration of music therapy had no effect on the measured oxytocin difference.

Finally, the analysis of the music therapist’s oxytocin values, i.e., the 28 averaged individual measurements, revealed a significant increase in the oxytocin levels [*p* = 0.006, Hedges’ g = −0.543 CI95% (−0.926; −0.150)].

With respect to the reliability of the results, the analysis of the 20% of samples measured in duplicate (*n* = 38) using a t-test for paired values showed a significant difference between the initial measurement and the control measurement (*p* = 0.007), as shown in Table 3. Even after excluding the four outliers (delta oxygen >30 pg./mL), there was still a significant difference between the first and second measurement. These measurement inaccuracies already indicate difficulties in using oxytocin as a biomarker in studies.

**Table 3:** Analysis of the 20% duplicated measurements (*n* = 38) using a paired samples *t*-test (total measurements: *n* = 192, including 32 children and the music therapist).

	*N*	Mean Oxytocin (pg/ml)	SD	95% CI	
				Lower limit	Upper limit
Measurement difference	38	−3.026	6.584	−5.190	−0.862

	T	Significance		Hedges‘correction [95% CI]
		One-tailed *p*	Two-tailed *p*	
Measurement difference	−2.833	0.004	0.007	−0.450 [−0.775; −0.120]

### Adherence

A total of 2 months recruitment time was required for this study and, after providing detailed information to parents and children, a consent rate of 100% (*n* = 32) was achieved for participation. There were no dropouts during the study, allowing it to be conducted as scheduled within 1 month.

However, there were some difficulties with protocol adherence in implementing the waiting list control group, as the children often had difficulty spending the full 30 min alone in a room. Consequently, several children occasionally left the room and interacted with other students and teaching staff during the time of the actual silent work. In this respect, a bias in the results of the control group cannot be ruled out. Similarly, the music therapy sessions could not always be carried out to its full extent, as the children did not always engage with it for the entire 30 min, so that the duration of the music therapy varied considerably (Table 1; Figure 5). Nevertheless, the study was well received by the students, and they cooperated to the best of their ability.

## Discussion

Overall, no significant change in oxytocin levels was measured either between groups, namely the music therapy and the waiting list control group, nor within groups. The non-significant increase in oxytocin concentration in the music therapy group, however, in line with previous literature (Fancourt et al., 2014; Grape et al., 2002; Kreutz, 2014), suggests a responsiveness of oxytocin to musical stimulation. In contrast, oxytocin levels remained unchanged in the waiting list control group.

When looking at Group 1 and Group 2 separately, music therapy showed a non-significant increase in oxytocin concentration in each respective group, whereas a slight decrease in oxytocin was measured in the control group of Group 1 and a slight increase in oxytocin in the control group of Group 2. Yet, a bias in the results of the control group cannot be ruled out with certainty due to the above-mentioned difficulties in the conduct of the silent work with these children. A similar problem was also evident in the implementation of the music therapy group, however, the evaluation showed that the considerably varying duration of the music therapy had no significant effect on the measured oxytocin difference. Nevertheless, an effect of the order of interventions is highly unlikely due to randomization to an allocation sequence. Further, a carryover effect is also not to be expected due to the short half-life of oxytocin and a washout phase of >24 h.

In the subgroup analysis, the results of the children with an autism spectrum disorder, who made up exactly half of the study population, were examined. No differences were found compared to children without autism spectrum disorder, so that no evidence of a specific response in oxytocin levels was found in the subgroup of children with autism spectrum disorder.

The music therapist showed a significant increase in oxytocin levels in the before-after comparison. This also demonstrated a clear responsiveness of the biomarker oxytocin to musical stimulation. Furthermore, the question arises as to whether repeated music therapy in particular is associated with an increase in levels and to what extent the perception of the situation, i.e., the feeling of well-being or discomfort during music therapy, has an influence on changes in oxytocin levels.

As far as we know, there is only one other study that measured oxytocin before and after music therapy, so there is little comparable data on this subject. Nevertheless, in this study by (Palumbo et al., 2022), also no significant changes in oxytocin levels before and after music therapy were reported. However, when looking at a wider range of studies, where oxytocin levels were measured before and after various types of music interventions, findings showed a responsiveness of oxytocin to music, but with contradictory results regarding an increase, decrease or unchanged oxytocin levels. The authors of the respective studies attribute the ambiguous results primarily to individual factors and contextual factors influencing the oxytocinergic system that were not sufficiently controlled or comparable in these particular studies (Bowling et al., 2022; Busse et al., 2024; Fancourt et al., 2016; Keeler et al., 2015; Schladt et al., 2017).

When considering oxytocin as a biomarker for attachment, there are therefore a series of challenges in interpreting the results. First of all, there are no standard values for oxytocin in a healthy normal population available in the literature (Martins et al., 2020), rather, human studies rely on the repeated assessment of oxytocin levels as pre-post measurements of an intervention, i.e., baseline values and changes in oxytocin levels, so that primarily an activity or reactivity of the oxytocinergic system to stimuli is investigated (Tabak et al., 2023). Further, against the background of contradictory results in oxytocin research the dependence of oxytocin on individual factors such as age, sex, female cycle as well as context is repeatedly pointed out (Bartz et al., 2011; Engel et al., 2019; Marazziti et al., 2019; Olff et al., 2013; Zak et al., 2022). Finally, interactions of oxytocin with other neuromodulator systems including dopamine, endorphine and serotonin makes the field even more complex and thus complicates the identification of neurobiological signaling pathways (Iovino et al., 2021).

In view of the growing literature on the topic of potentially reduced oxytocin levels in psychiatric patients, studies in which precisely this patient collective of children with adverse childhood experiences was examined with regard to oxytocin levels are of particular interest for the interpretation of the present study results. For example, in a study from Japan, Suzuki et al. (2020) showed that maltreated children had lower oxytocin levels compared to the healthy control group. In addition, Ellis et al. (2021) showed in a series of meta-analyses that adverse childhood experiences predicted lower oxytocin levels. Further research will thus need to address the role of adaptations of the oxtocinergic system in the context of early childhood experiences, as well as the potential for prevention and intervention.

Another field of oxytocin research is concerned with the genetics and epigenetics of the oxytocinergic system, including oxytocin receptor polymorphisms and methylation status (Harvey, 2020; Chen et al., 2020). However, the effects of oxytocin receptor alleles on social behavior are complex, assessments across studies are not always consistent, and the extent to which these inter-individual differences play a role in empathy, stress regulation and social behavior (Rodrigues et al., 2009; Sicorello et al., 2020) is still the subject of research.

Nevertheless, initial results are available with regard to the patient population investigated in this pilot study. For instance, in a meta-analysis, Poore and Waldman (2020) showed an association of oxytocin receptor polymorphisms and antisocial behavior. In addition, Moerkerke et al. (2021) in a systematic review showed that hypermethylation of the oxytocin receptor is associated with higher quantitative autism traits in adults. The transfer and inclusion of these results in the interpretation of the results of this pilot study is still challenging at this stage. Nevertheless, the field of genetics and epigenetics will be an integral part of oxytocin research in order to draw a comprehensive portrait of the complexity of the oxytocinergic system.

Taken together, this makes it particularly difficult to interpret results, namely the changes in oxytocin levels, of patients with different diseases or healthy controls and to classify their clinical relevance.

In addition, the measurement of oxytocin in different compartments of the body, namely CSF, plasma, saliva and urine, also poses a challenge, as various studies report correlations between the compartments (Valstad et al., 2017) and possible diurnal cycling of oxytocin levels, but there is no clear scientific consensus to date (Carson et al., 2015; Kagerbauer et al., 2019). Further, a lack of standardization in the measurement and evaluation of oxytocin samples is also repeatedly a subject of criticism, since results are often only comparable to a limited extent (Tabak et al., 2023).

Therefore, conclusions of the results should only be drawn with caution and against the background of the current state of knowledge in oxytocin research.

### Limitations

In terms of the limitations of this pilot study, the significance of the results obtained is limited due to the relatively small number of cases and should be considered preliminary. Due to the nature of the interventions, blinding was precluded and as the study design did not include a control group of healthy children, comparability with a healthy population is limited.

Finally, study participants were familiar with music therapy prior to the study and most of them already had received music therapy. This might have an impact on oxytocin measures given the potential for an already developed therapeutic relationship. However the study sample was too small to go into a detailed subgroup analysis including the participant’s history of music therapy. It is also unclear whether long-term music therapy modulates oxytocin response in humans or whether it remains to occur spontaneously, which is also discussed in a previously conducted systematic review on the role of oxytocin in music interventions (Busse et al., 2024). On the other hand, given these are children with social and emotional disorders, the struggle to develop attachment, a significant change in oxytocin within the timeframe of the study might also be questioned.

As already reported by (Chanda and Levitin, 2013), field studies such as the one presented here, suffer from confounding factors that relate primarily to the control intervention. In this study, too, adherence to the protocol was only partially possible in the waiting list control group as the study participants still acted with other children, which may have led to the non-significant results between the groups. On the other hand having children being alone in the control group makes the two conditions not comparable. A three-armed study containing these interventions (music therapy, social interaction, no intervention) would provide information on the corresponding effects, but was not feasible in the present setting.

Moreover, there is evidence from a recent meta analysis, that children with autism have altered oxytocin levels (John and Jaeggi, 2021). As half of the children were diagnoses with autism, this could this have affected the results. A *post hoc* analysis of the results however found no differences.

Another limitation is given by using salivary oxytocin levels as the primary and exclusive outcome measure. Therefore, the evaluation lacks data that take into account contextual aspects of the interventions that may influence the perception of the respective situation and thus the oxytocinergic system, e.g., the measurement of cortisol to control HPA axis activity (Schladt et al., 2017) or questionnaires on the children’s sense of well-being during music therapy and the control condition. In addition, due to the small sample size, the effect of potentially oxytocin-level altering medication such as methylphenidate (Levi-Shachar et al., 2020) was not taken into account in the present statistical analysis.

Overall, the particularity of the study population as well as the lack of control for contextual factors may therefore impede the generalizability of the results and the synthesis of findings across studies.

In addition, the evaluation of the 20% duplicate measurements in this pilot study revealed an inaccuracy in the measured values that proved to be significant. This demonstrates the importance of a larger study population for further research to detect an effect of music therapy on the oxytocinergic system.

Nevertheless, the use of saliva samples as a low-risk and cost-effective test offers a great opportunity to investigate the neuroendocrinological basis of music therapy non-invasively and without interrupting the therapeutic process. Future research will require larger studies including healthy controls to better investigate the neuroendocrinology of therapeutic alliance and thus attachment formation in music therapy. It will also require continued research into the oxytocinergic system to better understand individual and contextual factors influencing the oxytocinergic system. This combined knowledge may facilitate the interpretation of the reported changes in oxytocin levels and their clinical relevance, as well as enable the synthesis of findings across studies.

Overall, the importance of the oxytocinergic system for social behavior and its role in the development of diseases, but also as a target for therapy, is increasingly being recognized. The present study therefore aims to contribute by presenting music therapy as an attachment-based intervention that may influence the endogenous release of oxytocin and thus have an impact on social behavior.

Ultimately, it remains a difficult yet promising task to provide scientific neuroendocrinological evidence for music therapy.

## Conclusion

This pilot study described the basic feasibility of this study design and discussed the difficulties of conducting it with children with social and emotional disorders.

Although we found a difference between the music therapy group and the waiting list control group, this difference was not significant. The within group comparisons, namely the music therapy group, the waiting list control group and the autism spectrum disorder subgroup, also showed no significant changes in oxytocin levels. The music therapist, however, showed a significant increase in oxytocin levels in the before and after comparison. Yet, it remains unclear whether and to what extent changes in oxytocin levels are associated with clinical benefits. Larger studies with healthy controls and a better understanding of the oxytocinergic system as well as the role of individual and contextual factors for changes in oxytocin levels will be essential to better interpret the results obtained to date and their clinical relevance.

## Data Availability

The raw data supporting the conclusions of this article will be made available by the authors upon request.
